# Identifying species at coextinction risk when detection is imperfect: Model evaluation and case study

**DOI:** 10.1371/journal.pone.0183351

**Published:** 2017-08-28

**Authors:** Michaela Plein, William K. Morris, Melinda L. Moir, Peter A. Vesk

**Affiliations:** 1 School of Earth and Environmental Science, University of Queensland, St. Lucia, 4072, Australia; 2 School of BioSciences, The University of Melbourne, Parkville, 3010, Australia; 3 School of Biological Sciences, University of Western Australia, Crawley, Western Australia 6009, Australia; University of Florida, UNITED STATES

## Abstract

Losing a species from a community can cause further extinctions, a process also known as coextinction. The risk of being extirpated with an interaction partner is commonly inferred from a species’ host-breadth, derived from observing interactions between species. But observational data suffers from imperfect detection, making coextinction estimates highly unreliable. To address this issue and to account for data uncertainty, we fit a hierarchical *N*-mixture model to individual-level interaction data from a mutualistic network. We predict (1) with how many interaction partners each species interacts (to indicate their coextinction risk) and (2) how completely the community was sampled. We fit the model to simulated interactions to investigate how variation in sampling effort, interaction probability, and animal abundances influence model accuracy and apply it to an empirical dataset of flowering plants and their insect visitors. The model performed well in predicting the number of interaction partners for scenarios with high abundances, but indicated high parameter uncertainty for networks with many rare species. Yet, model predictions were generally closer to the true value than the observations. Our mutualistic plant-insect community most closely resembled the scenario of high interaction rates with low abundances. Median estimates of interaction partners were frequently much higher than the empirical data indicate, but uncertainty was high. Our analysis suggested that we only detected 14-59% of the flower-visiting insect species, indicating that our study design, which is common for pollinator studies, was inadequate to detect many species. Imperfect detection strongly affects the inferences from observed interaction networks and hence, host specificity, specialisation estimates and network metrics from observational data may be highly misleading for assessing a species’ coextinction risks. Our study shows how models can help to estimate coextinction risk, but also indicates the need for better data (i.e., intensified sampling and individual-level observations) to reduce uncertainty.

## Introduction

A major driver of biodiversity loss is secondary extinction or coextinction, which is the loss of a one species resulting from the loss of an interaction partner. Due to the significance of coextinctions, it is important to estimate how many species are at risk. Until now, coextinction risk estimates were based on the strength of interactions between species using museum records (e.g., [1]), presence-absence data (e.g., [2, 3]), or species interaction networks from empirical studies [4]. Interaction data has also been increasingly used to investigate the structure of communities [5], their stability [6], their function [7] and the role of individual species in networks [8]—factors that are thought to influence coextinction risk.

Like any other observational data, interaction data are prone to sampling bias, which results from limited sampling effort, imperfect detection of species and other sources of variability [9, 10]. Species may remain undetected when they are cryptic, rare or when their phenology and study period misalign. Interactions between species are even harder to detect than the species themselves [11]. Species abundance has been shown to explain most of the variation in network metrics [12, 13] and predicts various network metrics such as connectance, nestedness, interaction evenness, and interaction asymmetry [12]. Network metrics that are commonly used to assess the structure and stability of interaction networks and the coextinction risk of species are degree distribution, network specialisation $H_{2}^{\prime }$, weighted nestedness and modularity [14]. Further, species abundances and community composition vary spatially [15] and temporally [16], causing species to appear as specialists in one study season and as generalists in another [17]. Thus, observed interaction networks are biased towards abundant species and hence inaccurate representations of true networks.

Although imperfect detection has been widely acknowledged in species occupancy models and other biodiversity studies (e.g., [9, 18]), analyses of interaction networks largely ignore the problem ([19, 20], but see [21–23]). Some studies have attempted to overcome the problem of imperfect sampling by increasing sampling intensity. These studies show that detecting species and links accurately requires intensive sampling effort [11, 24]; even after four years of intensively sampling a pollinator community, new interactions and species continued to emerge [17]. Uncovering the true network by increasing sampling effort is costly [24], and for species-rich habitats, probably infeasible [9, 18]. To mitigate imperfect sampling effects in interaction networks, network analysis has, therefore, turned towards modelling approaches, including null models [25] and quantitative niche models [23]. These approaches show that sampling intensity influences most network metrics, in particular those related to the specialisation of species [22, 23, 25, 26]. Despite the importance of uncertainty in ecology (e.g., [27]) and conservation [28], none of the studies have reported uncertainty in network metrics.

Uncertainty in network data arises for several reasons. Firstly, interaction frequencies are mostly condensed into species-by-species matrices, which leads to the loss of crucial information about the variability of interactions across individuals, and confounds abundances and interaction preference [21]. Some have tried to disentangle abundances from species preferences, by assessing the eigenvalues of species adjacency matrices to identify system stability (i.e., negative eigenvalues identify stable systems) [29]. Yet, they did not go as far as to look at the distribution of interactions among individuals.

Secondly, uncertainty in interaction networks arises from the natural excess of zero-observations [19]. Zeros occur when a particular species pair does not interact (true negatives), and due to imperfect sampling (false negatives, [30]). We can deal with this zero-inflation in the data by maintaining the original data structure (on the level of observed individual organisms) and analysing them with hierarchical, mixture models [30]. Studies have applied mixture models to estimate uncertainty in network data, revealing considerable uncertainty in predictions of network specialisation [21] and host-specificity [31]. Although some studies have predicted that sampling effort does indeed influence network metrics [21, 23], no study has ever evaluated how uncertainty changes under varying interaction probabilities and species abundances.

Here, we investigate how variations in sampling effort, interaction probability between plant and animal species, and the abundance of animal species influence the inferences from mutualistic interaction networks. Specifically, we are interested in how these parameters influence estimates of 1) the number of mutualistic interaction partners of each species and 2) the total species richness of the animal community. We fit a model to individual-level interaction data to estimate two parameters: the probability that a mutualistic interaction between a plant individual and an animal species occurs, and the abundance of the animal species. We then use these parameters to estimate the number of interaction partners for each species, and the overall species richness of animals, including those species that are missed by the observation process. We apply the model to simulations and an empirical pollinator data set. By addressing imperfect detection and sampling completeness in such a framework, we ultimately derive coextinction risk estimates that better capture the uncertainty in our predictions.

## Material and methods

Here we assessed the question of how bias in interaction network data influences coextinction risk estimates. Although we focused on mutualistic interactions between plant and animal species, the same principal applies to antagonistic systems, such as herbivore-plant networks. Our method included several steps:

1. First, we developed a model drawing upon two previously published hierarchical models: one predicts the host plant use of herbivore insect species [31] and another that accounts for observation bias by estimating the probabilities of occupancy and detection [18]. Both models are Bayesian and account for uncertainty in parameter estimates.
2. Our model contained two submodels: one for the probability that two species interact (hereafter interaction probability) and another that estimated the abundance of animal species.
3. The interaction probability allowed us to predict the number of partner species that each species interacted with and the species richness of the animal community.
4. We tested the performance of our model on twelve simulated networks. The simulations had different values for the following parameters 1) interaction probability, 2) animal abundance and 3) sampling effort.
5. Finally, we applied the model to an empirical interaction network of flower-visiting insect species on a threatened community of flowering plants.

### The model

For each network, we modelled a matrix of interaction frequencies *Y*_*ij*_ between plant individual *i* and animal species *j* as random realisations of a zero-inflated Poisson distribution. The interaction matrix elements consist of discrete, non-zero interaction frequencies between animal species and plant individuals, and of zeros where no interaction was observed. Thereby, we preserved information about the spread of interactions of an animal species across all sampled individuals of a plant species. This contrasts with conventional bipartite network analyses, where interaction frequencies are aggregated in species by species matrices [21], and allows us to estimate uncertainties in interaction frequency [31]. To deal with the large amount of unobserved plant-animal interactions, we applied a zero-inflated *N*-mixture model [32]. Since we were further interested in how well a network study captures the species richness of the animal community, we applied a method from occupancy and detection studies; a method initially developed to estimate the number of undetected species in a community based on temporally replicated counts of species at different sampling sites [18]. Combining the two approaches allowed us to estimate the number of available, but unobserved, animal species, and thereby the total number of species in the community.

In our model, interaction frequencies *Y*_*ij*_ were modelled as a function of two parameters: 1) an indicator variable *θ*_*k*[*i*]*j*_ that described if an interaction between an animal species *j* and a plant species *k* (that plant individual *i* belongs to) occurred and 2) the animal species ‘abundance λ_*j*_.

$$\begin{array}{c} Y_{ij}∼Poisson\left(\theta_{k[i]j}\cdot \lambda_{j}\right) \end{array}$$ (1)

The interaction frequencies *Y*_*ij*_ were conditional on the occurrence of an interaction between plant species *k* and animal species *j*. The interaction indicator *θ*_*k*[*i*]*j*_ took values of either 1, for plant-animal pairs that interact, or 0 when no interaction occurred. The modelling framework was hierarchical because the two parameters, the interaction indicator *θ*_*k*[*i*]*j*_ and the animals’ abundance λ_*j*_ are derived from distributions with their own hyperparameters, drawn from overarching hyperdistributions [33].

### Modelling animal abundance

We estimated the abundances of an animal species λ_*j*_ in a Poisson regression with an intercept term for the average abundance *β*_0_ across all animal species and a random effect coefficient *β*_1*j*_ for a particular animal species *j*.

$$\begin{array}{c} \log\left(\lambda_{j}\right)=\beta_{0}+\beta_{1j}. \end{array}$$ (2)

Hence, we modelled the expected abundance of an animal species *j* regardless of which plant species it interacted with. Varying the abundance with each plant species would have been possible, but would have hampered model fitting and increased computational costs substantially. Further, empirical mutualistic networks are usually obtained by observing an (immobile) plant individual and sampling all its (mobile) animal visitors, making it difficult to collect information about the movements of individual animals or (as in our study) even impossible when animals are collected for later identification in the lab.

### Modelling interactions between animal and plant species

The interaction indicator *θ*_*k*[*i*]*j*_ derived from a Bernoulli trial with an interaction probability Θ_*kj*_ between an animal species *j* and a plant species *k*.

$$\begin{array}{c} \theta_{k[i]j}∼Bernoulli\left(\Theta_{kj}\right). \end{array}$$ (3)

We derived the interaction probability Θ_*kj*_ from a logistic regression with a probability that an animal species interacted with a plant species, *ψ*_*kj*_, and a latent variable *ω*_*j*_. This latent variable *ω*_*j*_ indicated if an animal species occurred in the community or not and was randomly drawn from a Bernoulli distribution. We modelled the interaction probability between a plant species and an animal species, *ψ*_*kj*_, with a linear regression including an intercept term *α*_0_ for the average interaction probability of the animal species *j* across a plant species *k*, and a (random) effect coefficient *α*_1*jk*_,

$$\begin{array}{c} \text{logit}\left(\Theta_{kj}\right)=\psi_{kj}\cdot \omega_{j}, \end{array}$$ (4)

$$\begin{array}{c} \omega_{j}∼Bernoulli\left(\Omega_{j}\right), \end{array}$$ (5)

$$\begin{array}{c} \psi_{kj}=\alpha_{0}+\alpha_{1kj}. \end{array}$$ (6)

Vesk et al. (2010) assumed that interaction probabilities are influenced by phylogenetic relationships (i.e., an animal species had a higher chance of interacting with plant species of the same family). While such relationships are certainly possible (e.g., [34]), we did not include it here because the plant species in our case study belong to very few families (one in 2012 and three in 2013). One could also assume that a plant species is visited more often by dependents of the same family, but we omitted such a potential relationship due to ambiguous evidence in the literature (e.g., [35]).

Similarly to Dorazio et al (2006), who predicted species richness in a community of birds and butterflies, we estimated the unknown species richness *N* of animals in our case study. When the total number of species is unknown, the Markov Chain Monte Carlo (MCMC) approach struggles to fit the model because the dimensions of the parameter vectors change with each draw of the parameter *N* [18]. We therefore augmented the observed community of animal species *n* with an arbitrary (but large enough) number of unobserved species *m* to create a “supercommunity” *S*, with fixed dimensions. The species richness *N* was not directly estimated as a parameter, but indirectly as the sum of all available species *ω*_*j*_.

$$\begin{array}{c} N=\sum_{j=1}^{S}\omega_{j}. \end{array}$$ (7)

To check if our model was sensitive to the size of the supercommunity, we fitted it to the sampled networks of the HILA scenario with low sampling effort and checked for three different supercommunity sizes: 250, 300 and 450 species. A different size for the supercommunity did not affect the parameter estimates (see S1 Fig), but we have to ensure that the supercommunity is large enough to contain the estimates community size.

To estimate the number of interaction partners for each species (i.e., the number of plant species for each animal species *NP*_*j*_ and the number of animal species of each plant species *NA*_*k*_), we summed the interaction indicator *θ*_*kj*_ for each animal species *j* across all its plant species *k* and vice versa for each plant species across all animal species.

$$\begin{array}{c} NP_{j}=\;\sum_{k}\theta_{kj}, \end{array}$$ (8)

$$\begin{array}{c} NA_{k}=\;\sum_{j}\theta_{jk}, \end{array}$$ (9)

### Bayesian model fitting and prior specifications

We fitted the model in a Bayesian setting with vague prior distributions for all model parameters. The regression coefficients *α*_1*kj*_, which modelled variation in the interaction probability between plants and animals, were drawn from a normal distribution centred on zero with a standard deviation *σ*_*α*_ for the variation among plant-animal pairs. Similarly, the variation in the average abundance among animal species *β*_1*j*_ was drawn from a normal distribution with a mean zero and a standard deviation *σ*_*β*_. We specified half-Cauchy priors for the standard deviations *σ*_*α*_ and *σ*_*β*_ [36] which we truncated at a lower bound of zero, *σ* ∼ half-Cauchy(0, 10). As per Eq 5, *ω*_*j*_ were randomly drawn from a Bernoulli distribution, with hyperparameter *Ω* ∼ *U*(0, 1). We used MCMC sampling to estimate the posterior probability distributions of parameters using rjags [37] in R [38]. For simulated data, we modelled four chains with 200,000 iterations each and checked for model convergence. For observed data, we ran four chains each for 1,000,000 iterations with a burn-in of 100,000 and a thinning rate of 100. We discarded the first 10,000 iterations as burn-in and thinned the chains by a factor of 100. If some parameters were not converged, we updated the chains for another 200,000 iterations with a thinning rate of 100 and no burn-in. We checked for convergence visually by examining the four chains, and analytically with the *Rhat* statistic. Parameters were judged to have approximately converged below an *Rhat* = 1.1 [36]. We provided the code for the Bayesian model in the supplement (S1 Code).

### Simulation study

We simulated twelve interaction network scenarios to explore how different parameterisations affected model performance and uncertainty. These networks contained ten plant species and 150 animal species. The parameter scenarios were a combination of low and high values for the average interaction probability and the average animal abundance, and three different sampling sizes. The values of these parameters were chosen to encompass a wide parameter space (Table 1). In *low-interaction* scenarios, we assumed an interaction to occur in approximately 1% of species pairs, whereas in *high-interaction* scenarios, an interaction was assumed to occur in approximately 80% of species pairs. In *low-abundance* scenarios, one animal occurred in approximately 20 observations, whereas in *high-abundance* scenarios there was approximately one animal in every observation. We sampled from each simulated network with three different sampling intensities with 20, 40 and 60 samples per plant species. The lowest sampling intensity represents a medium to high sampling effort in a field study (around five observation hours per species; e.g., [24]). Sampling intensities of 40, or even 60 are unusual for empirical studies. We chose these efforts here to investigate the required sampling size for interaction networks.

**Table 1:** Values for the mean and standard deviation of the two hyperparameters, the interaction probability and the abundance.

			abundance (log scale)		
			high 0	low -3	sd
interaction probability (logit scale)	high	1.5	high interaction, high abundance	high interaction, low abundance	1.5
	low	-2.5	low interaction, high abundance	low interaction, low abundance	
	sd		2.5		

With these different parameter combinations and three different sampling intensities, we simulated twelve scenarios of interaction networks between animal species and plant individuals. We generated hyperparameter distributions for each interaction probability between a plant-animal pair and an animal species’ abundance from predefined distributions. We used these hyperparameters as scale parameters for sampling binary interactions from binomial distributions, and expected abundances of animals from Poisson distributions. The observed interaction frequencies between each plant individual and animal species are the product of the binary interaction and the expected counts. We provided the code for these simulations in the supplement (S2 Code).

### Evaluation of model performance

Firstly, we evaluated how well our model recovered the simulated data by visually comparing simulated parameters (“true”) with model predictions (“estimated”) for the following parameters:

- the interaction probability,
- the realised interactions between a plant and an animal species, and
- the abundance of animals.

We calculated receiver operator statistics to assess the performance of the binary interaction parameter and summarised these with the area under the curve statistic (AUC) to compare the performance of the model for different parameter scenarios (see S1 Table).

To evaluate how well the model predictions reflected the simulated networks, we compared:

- the number of interaction partners of each plant and animal species, and
- four common network metrics (degree distribution, specialisation $H_{2}^{\prime }$, weighted nestedness and modularity)

between simulated networks (“true”), networks that were sampled from simulations (“sampled”), and networks that were predicted by the model (“predicted”). To account for parameter uncertainty, we generated 1,000 random networks based on the model predictions.

### Network metrics

Several network metrics are commonly used to assess the structure and stability of interaction networks: degree distribution, specialisation $H_{2}^{\prime }$, weighted nestedness and modularity [14]. For simulated and sampled networks, we were only able to calculate point measurements, while for the 1,000 predicted networks, we report mean and 95% confidence intervals for each metric. We compare the metrics of simulated, sampled and predicted networks to check how sensitive network metrics are to imperfect detection and sampling effort.

The number of links of a species is the species’ degree and the degree distribution reflects the probability distribution of all degrees over the whole network [39]. Animal-plant mutualistic networks often exhibit truncated power-law distributions: some species have many interaction partners while most species only interact with few others [39].

Nestedness measures the departure of a matrix from being perfectly nested. Species interact with a subset of those species that the most linked interaction partner interacts with [25]. Since networks are described not only by their topology but also by their differences in the frequency of interactions, we calculated the weighted version of nestedness (WNODF) for quantitative networks. WNODF is based on overlap and decreasing number of interactions [40] and shows values range from 0 (not nested) to 100 (highly nested).

The number of interaction partners per species can be a measure of a species’ coextinction risk. We calculated network specialisation ($H_{2}^{\prime }$) to see how many of the potential network interactions were realised [41]. Network specialisation $H_{2}^{\prime }$ combines the specialisation of species across the entire community and thereby quantifies the niche redundancy of animal species across different plant species, and vice versa [41]. It ranges from 0 (no specialisation) to 1 (perfect specialisation) [25].

Finally, we calculated the modularity of networks. Species in animal and plant networks have been found to interact mainly with other species within the same module thereby forming subnetworks of interacting species [42]. Modularity measures how compartmentalised a network is and may have a slight stabilising effect on networks [42]. Recently several algorithms have been proposed to detect modularity in quantitative interaction networks. We used the LPAwb+ algorithm because it is the fastest for large networks [43].

### Case study: Observed interactions in a community of flower visitors and their plant plants

We observed flower-visiting insect species on a community of threatened plants from the Stirling Ranges National Park in south-west Australia (S2 Fig). The region is species-rich with approximately 3,600 endemic plant species, of which 1,909 are threatened [44]. Due to the high numbers of endemic plant species within the region, and the level of anthropogenic disturbance, it was identified as one of the original hotspots of biodiversity [45]. There are potentially many threatened invertebrate species associated with the threatened flora [46]. Several plants in our focal community are managed because they are highly threatened. Managers are also interested in identifying those insect species that have a high chance of coextinction.

The observed plant community consisted of a total of eleven plant species (six in 2012 and ten in 2013) mainly from the family Ericaceae, but also from the Myrtaceae and Fabaceae (Table 2). The focal species are part of the “Eastern Stirling Range Montane Heath and Thicket Community”—a plant community identified as critically endangered under the International Union for the Conservation of Nature’s Red List Criteria for Ecosystems [47]. This dense shrub thicket community is only found on the highest peaks of the Stirling Ranges, which are distinctively montane with skeletal organic soils, low temperatures, high humidity and light exposure, and occasional snowfalls [46].

**Table 2:** Observed plant species and the number of observed individuals per species and study year.

Family	Genus	Species	2012	2013
**Ericaceae**	*Andersonia*	axilliflora*	27	56
	*Andersonia*	*echinocephala*	28	46
	*Andersonia*	sp. “Stirling Range”	4	2
	*Dielsiodoxa*	*tamariscina*	32	–
	*Leucopogon*	*atherolepis*	–	8
	*Leucopogon*	gnaphalioides*	101	56
	*Sphenotoma*	sp. “Stirling Range”	49	40
**Myrtaceae**	*Calothamnus*	*crassus*	–	16
	*Kunzea*	*montana*	–	35
	*Taxandria*	*parviceps*	–	33
**Fabaceae**	*Aotus*	*genistioides*	–	37

*critically endangered species

Two plant species within the community, the Stirling Range Beard Heath (*Leucopogon gnaphalioides*) and the Giant Andersonia (*Andersonia axilliflora*), are critically endangered under both Australian state and federal legislation because their naturally small populations are threatened by increasing recreational activity, disease (*Phytophthora cinnamonii*) and wildfire [48, 49]. The few remaining populations are restricted to only a few mountain peaks 900 m above sea level [48, 49]. The largest population occurs on Bluff Knoll, the highest peak of the mountain range at 1090 m above sea level [48]. We restricted our study to the population on Bluff Knoll, because it was the largest and most accessible.

In 2012, we observed the two focal plant species and four non-threatened plant species in the same family (family Ericaceae, Table 2). In 2013, we broadened our observations to include three additional species, which were important flowering plants in the previous year (Table 2). We did not repeat sampling on *Dielsiodoxa tamariscina*, because we did not detect visitors in 2012 other than one highly abundant ant species. *L. artherolepis* was not detected flowering in 2012, but a few individuals were found in 2013. Each study season started slightly before and ended slightly after the peak flowering time of the two focal species, which was strongly dependent on climatic conditions (4th—17th December 2012, 22nd November—10th of December 2013).The number of observed plant individuals differed every year due to availability (Table 2). The observation time was standardised to 15 min for each plant individual. We captured all insects that we detected foraging on the focal plant‘s flowers. Between observations we moved to the next plant and allowed 5 min for potentially disturbed insects to return to the flowers, before starting another observation. We only conducted our sampling on dry days with a temperature above 15°C, because insect activity was extremely low in wet and cold conditions.

## Results

### Model performance

To evaluate how well the model recovered the interaction and abundance parameters, we compared simulations with their predicted counterparts *θ*_*kj*_ and *β*_1*j*_. Estimated abundances of animal species *β*_1*j*_ most closely resembled true abundances in the HIHA scenarios (S3a–S3c Fig). For *low-abundance* scenarios (HILA and LILA), abundance estimates exhibited a wider spread than in *high-abundance* scenarios (S3g–S3l Fig). The simulation generated animal species without any plant interactions in each but the HIHA scenarios. Animals without plant interactions were assumed to have abundances equal to the modal estimates, which lead to the horizontal lines of points (with large deviations) in the LIHA and LILA scenarios (S3d–S3f and S3j–S3l Fig).

We also compared simulated binary interactions with the mean estimated interactions. This revealed increasing model accuracy for larger sampling efforts in *high-abundance* scenarios (HIHA and LIHA). For lower sampling efforts in HIHA and LIHA scenarios, unobserved interactions (i.e., false positives) were estimated to occur with a probability of 0.38 (S4a and S4d Fig). For high observation effort all true interactions and true non-interactions were correctly estimated (S4c and S4f Fig). In *low-abundance* scenarios (HILA and LILA), the model struggled to predict some non-interactions and interactions correctly (S4g–S4l Fig): some true non-interactions were falsely identified as interactions with a mean probability of up to 0.61 in HILA and 0.15 in LILA scenarios. In contrast, some true interactions showed mean estimated probabilities of 0.01-0.60. In the HILA and LILA scenarios, increasing the sampling effort had little effect on the accuracy of model estimates.

The receiver operator statistics, summarised in the area under the receiver operator curve (AUC) values were close to 1, which indicated that the model performed well in correctly identifying the simulated true positives and had a comparatively low rate of false positives (S1 Table). We received higher AUC values with increasing sampling effort, indicating improved performance with increasing data availability.

### Number of interaction partners in simulated networks

**Fig 1:**
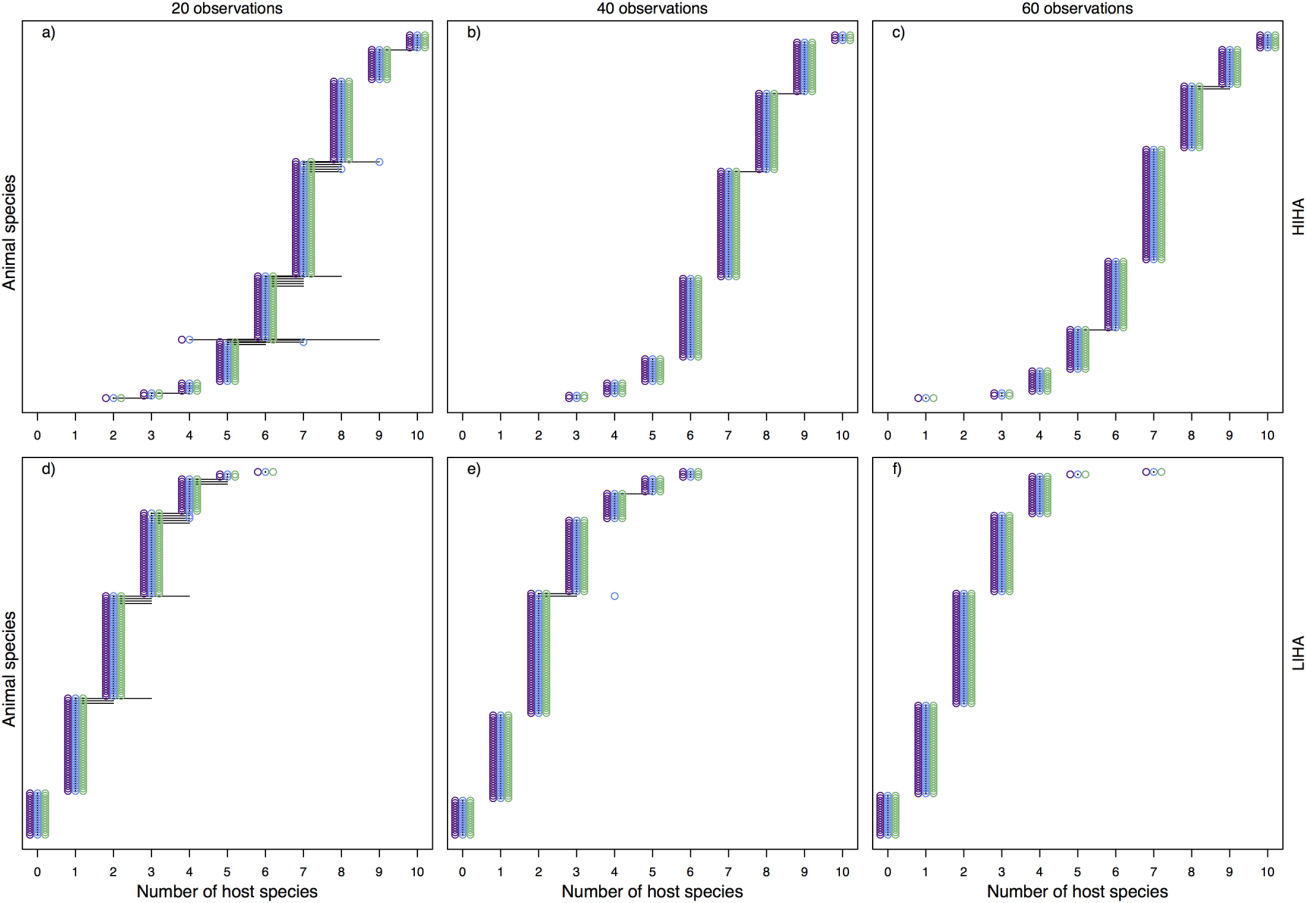
Number of plant partner species of each animal species in *high-abundance* scenarios (HIHA a-c and LIHA d-f). Blue represents the true number of plant partner species, purple shows the sample and green gives the median estimates. Thick and thin black lines give the 50% and 95% credible intervals, respectively. To improve readability, we have jittered the circles slightly (observed slightly to the left, estimated slightly to the right), which causes the points to appear to be away from their discrete number.

**Fig 2:**
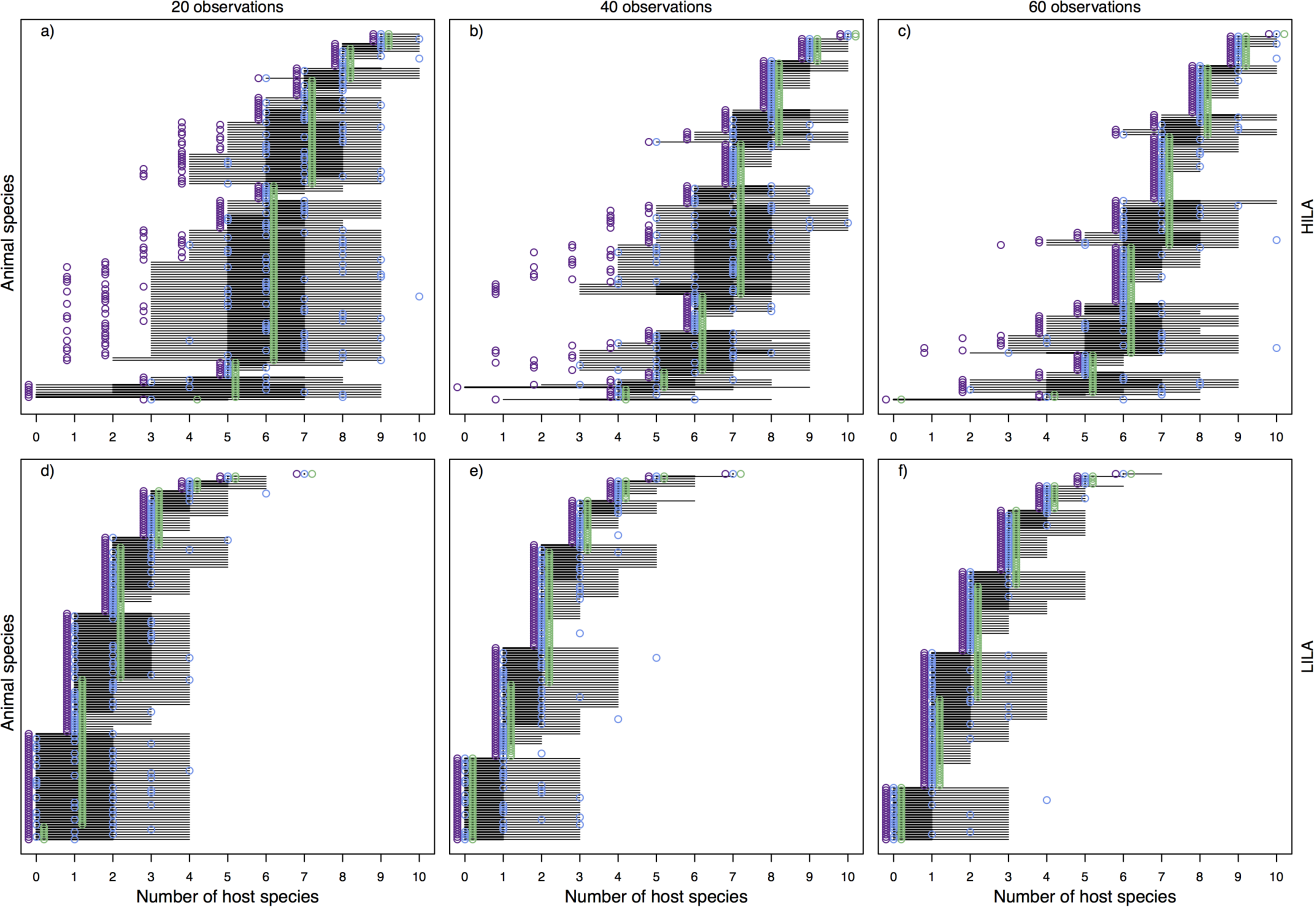
Number of plant partner species of each animal species in *low-abundance* scenarios (HILA a-c and LILA d-f). Blue represents the true number of plant partner species, purple shows the sample and green gives the median estimates. Thick and thin black lines give the 50% and 95% credible intervals, respectively. To improve readability, we have jittered the circles slightly (observed slightly to the left, estimated slightly to the right), which causes the points to appear to be away from their discrete number.

**Fig 3:**
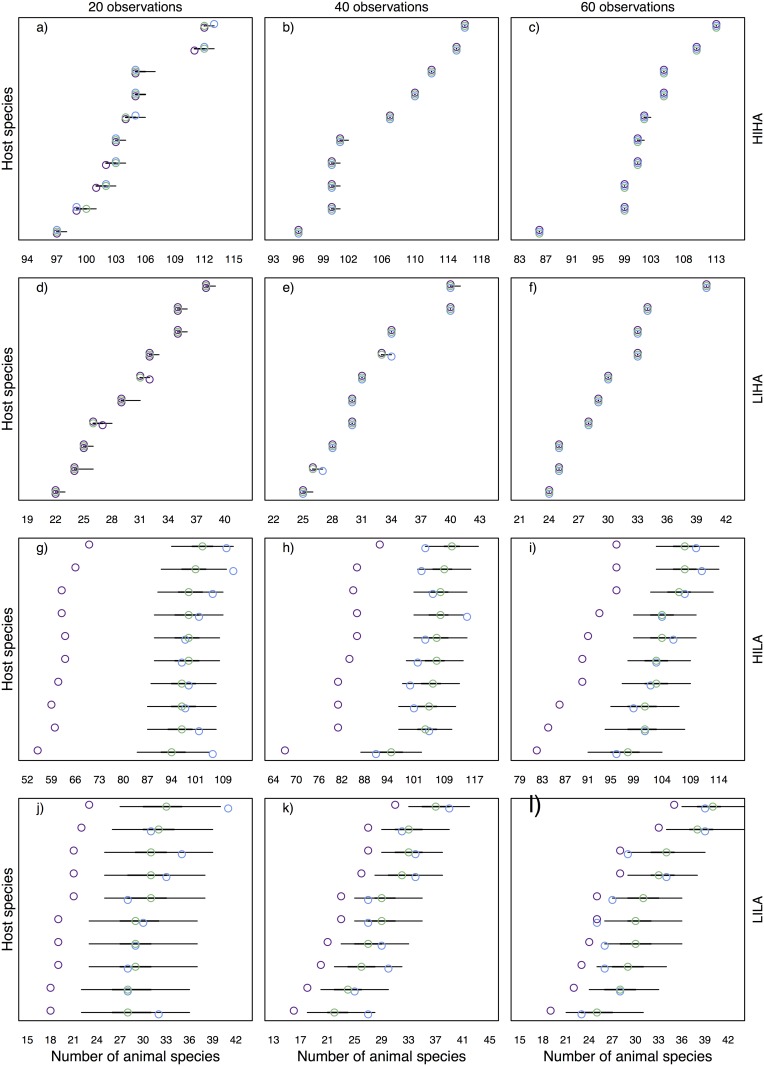
Number of animal partner species of each plant species in simulated scenarios (HIHA a-c, LIHA d-f, HILA g-i, LILA j-l). Blue represents the true number of plant partner species, purple shows the sample and green gives the median estimates. Thick and thin black lines give the 50% and 95% credible intervals, respectively.

We compared model estimates to simulated (true) numbers of interaction partners and to sampled numbers to evaluate model performance and verify whether estimates represented an improvement over the observations. Overall, our model predicted the number of interaction partners (i.e., diet breadths) of species as well as, or better than simple observations (Figs 1, 2, 3).

For high abundances (HIHA and LIHA), the information in sampled interactions network is adequate for estimating the number of interaction partners and the model did not provide a great improvement over the sampling, even at low sampling sizes (Figs 1 and 3a–3f). The model underestimated the true number of interaction partners only in a few cases for low observation efforts (Fig 1a and 1d), yet it was still captured within the credible interval. When simulated abundances were low, our model performed better in predicting the number of interaction partners than the sampling and included the true value in its uncertainty range in most cases. The median estimates represented a better approximation than sampled values, especially in HILA scenarios (Fig 2a–2c). If true values differed to median estimates they were mostly captured in the 95% credible interval, and estimates became more certain with increasing sampling effort.

For the number of animal species that each plant species interacted with, we retrieved similar patterns. In *high-abundance* scenarios, true sampled and estimated values were closely aligned (Fig 3a–3f). In *low-abundance* scenarios, the estimates represented a much better approximation of the true values than the sampled values (Fig 3g–3l). The sampled values were furthest from the true values in the HILA scenarios (Fig 3g–3i). As before, the uncertainty around median estimates decreased with increasing sampling effort.

### Network metrics

The degree distribution for simulated, sampled and predicted interaction networks exhibited scenario-specific responses. While simulated, sampled and predicted degree distributions exhibited similar trends in high interaction scenarios (HIHA and LIHA), the model provided better estimates when interaction rates were low. Increasing the sampling size decreased the uncertainty around predicted degree distributions of plant species especially for low interaction rates and slightly for high interaction rates (S5 Fig). At low interaction rates, sampled degree distributions consistently decayed much faster than degree distributions from simulated and predicted networks.

**Fig 4:**
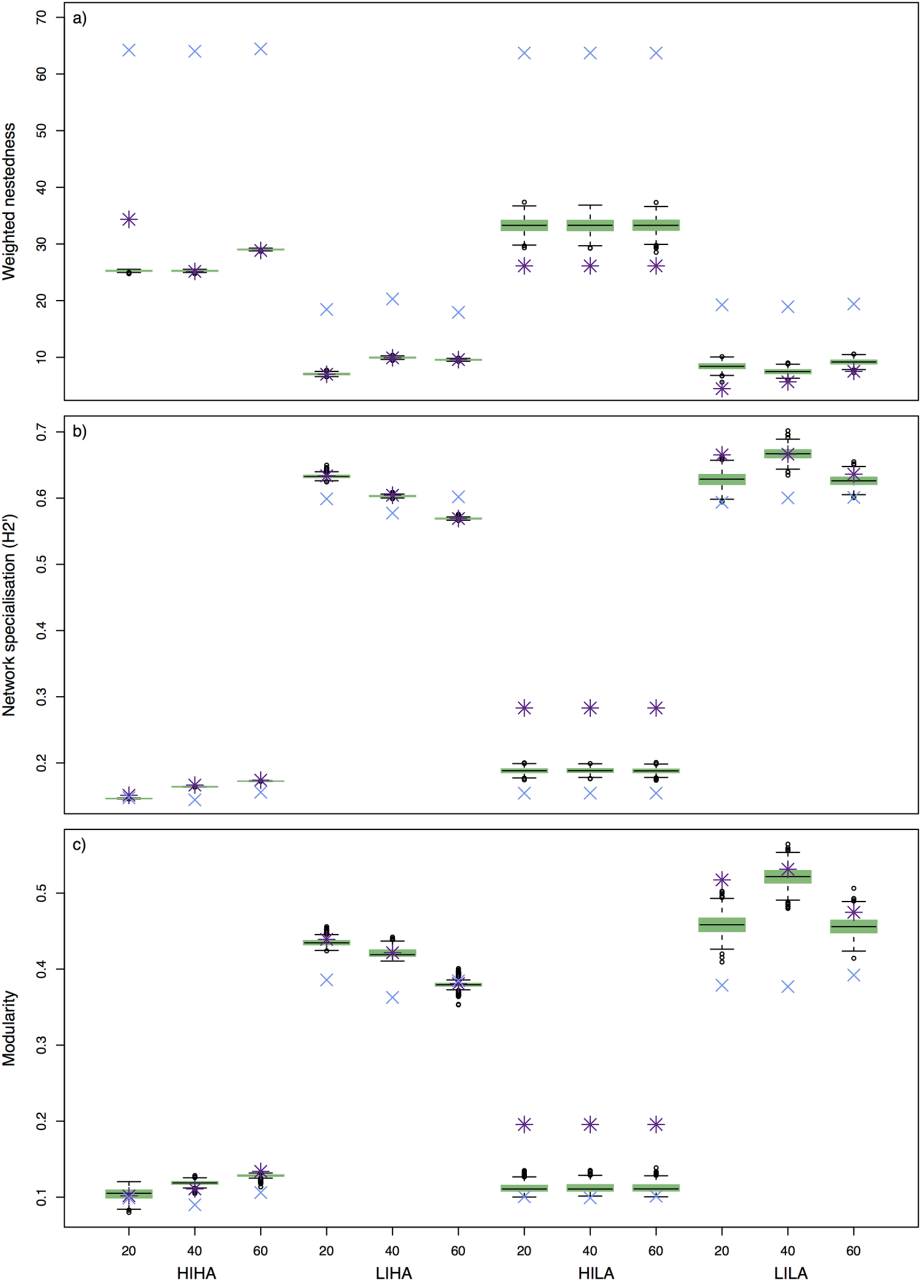
Network metrics for simulated networks. Network metrics a) weighted nestedness, b) network specialisation and c) modularity for simulated networks (blue cross), networks sampled from simulated data (purple star), predicted and sampled networks for different parameter scenarios (green boxplot). Boxplots give the median (black line), the interquartile range (green box) and the range of 1.5 of the interquartile box (whiskers) for predicted networks.

WNODF of simulated networks was high for scenarios with high interaction rates (∼65) and lower for scenarios with low interaction (∼20, Fig 4a). Sampled and predicted WNODF was always lower than simulated data: ∼25-35 for high interaction and ∼5-10 for low interaction. While we found no difference between sampled and predicted WNODF in high abundance scenarios, predicted metrics were often closer to the simulation than the observation in low abundance scenarios.

Network specialisation $H_{2}^{\prime }$ ranged from ∼0.17 for simulated *high-interaction* scenarios to ∼0.4 for *low-interaction* scenarios (Fig 4b). The specialisations of sampled and predicted networks were very close to the simulations only for HIHA scenarios, but furthest for HILA scenarios. In the latter case the predictions served as a considerable improvement over the observations, which were around 0.1 more specialised than the simulated dataset. Modularity of quantitative networks showed very similar patterns to network specialisation: high interaction scenarios showed a low modularity, whereas modularity was high when interactions were low (Fig 4c). As for network specialisation, observed and predicted metrics were very close to the simulation for *high-abundance* scenarios and overlapped for all but the HILA scenarios. In the latter case the predictions again exhibited an improvement over the observation. The uncertainty around the estimated metrics was low in HIHA and LIHA scenarios and relatively small in HILA and LILA scenarios. Increased sampling effort did not lead to improvements in recovering simulated metrics.

### Case study: The flower visitors of a threatened ecological community

We observed insect visitations at 241 plant individuals in 2012 and 329 plant individuals in 2013, resulting in 60.25 and 81.5 hours of observation, respectively. In 2012, we sampled 217 foraging insects representing 41 species, and in 2013 we sampled 948 foraging insects from 105 species. While Coleoptera and Hymenoptera were the most abundant and species-rich orders in both years, Diptera were also highly represented in 2013. The most abundant species in 2012 was the true bug *Spilostethus pacificus* (Hemiptera, Lygaeidae) with 30 individuals. In 2013 the European honey bee, *Apis mellifera* (Hymenoptera, Apidae) was highly abundant, closely followed by a native bee species, *Lasioglossum* sp26 (Hymenoptera, Halictidae).

The parameter predictions for the case study were most similar to the HILA scenario, with interaction probabilities of 0.40 (hereafter 95% credible intervals appear in brackets thus: (0.18, 0.65) and 0.55 (0.17, 0.48) in 2012 and 2013, respectively. The standard deviation of the interaction probability, *σ*_*α*_, was large and right skewed, 0.999 (0.5, 1) in 2012 and 0.996 (0.5, 0.999) in 2013. Model estimates of mean abundance *β*_0_ were very low with 0.0009 (0.0004, 0.002) in 2012 and 0.002 (0.001, 0.004) in 2013, corresponding to a mean abundance of one animal species in 445-2850 observations in 2012 and one in 235-775 observations in 2013. The standard deviation of the abundance *σ*_*β*_ was normally distributed around a value of 10.91 (7.39, 16.12) in 2012 and 9.58 (7.24, 12.68) in 2013.

The augmentation of empirical networks with 250 additional insect species resulted in supercommunities of 291 species in 2012 and 355 species in 2013. The model predicted a mean total species richness of 179 (85, 285) in 2012 and 262 (178, 349) species in 2013 (S1 Table). Since, the credible interval did not include the maximum available number of species, the actual community is likely to be smaller than the supercommunity.

### Comparison of Stirling Range networks and network simulations

**Fig 5:**
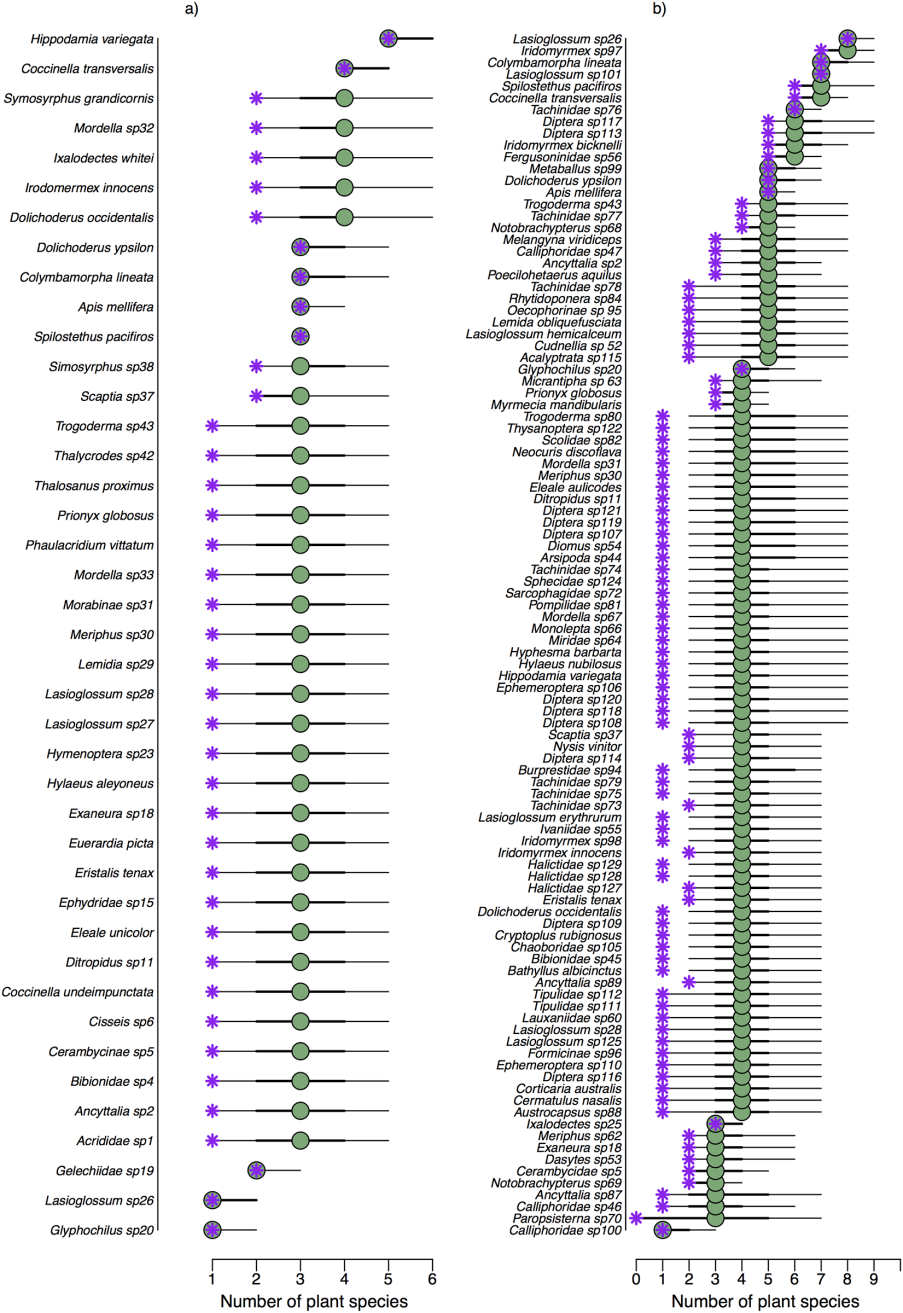
Number of plant species per insects species in the Stirling Range case study. Purple stars indicate observations and green circles represent median estimates. Thick and thin black lines give 50% and 95% credible intervals, respectively.

The number of plant species that each insect species interacted with appeared most similar to the HILA scenario for low sampling effort. While the observed number of plant species was always lower than the median estimates their ranges are similar, ranging from one to five plant species per insect in 2012 and zero to eight in 2013 (Fig 5). Yet, observed numbers of plant partner species were mostly located at the lower end of the 95% credible interval, with one to three plant species per animal less than estimated (Fig 5). The 95% credible intervals of estimated plant partner species were large for most species (Fig 5)—one to five plant partner species in 2012 and one to seven in 2013—implying that estimates were very uncertain for some insect species. Other species, however, exhibited very narrow uncertainty ranges; for example, *Spilothethus pacificus*, the most abundant flower-visitor in 2012, was predicted to interact with three plant species (3.0, 3.0). Likewise, in 2013 *Lasioglossum* sp.101 was observed to interact with seven plants and its median estimate confirmed this. Some species, such as the native bee *Lasioglossum* sp26 exhibited considerable temporal variation: while it was only observed and predicted to interact with one plant species (1.0, 2.0) in 2012, its observed and median number of plant partners was eight (8.0, 9.0) in 2013. The observations and median estimates aligned for only nine insect species in 2012 (from a total of 41) and ten insect species in 2013 (from 105).

**Fig 6:**
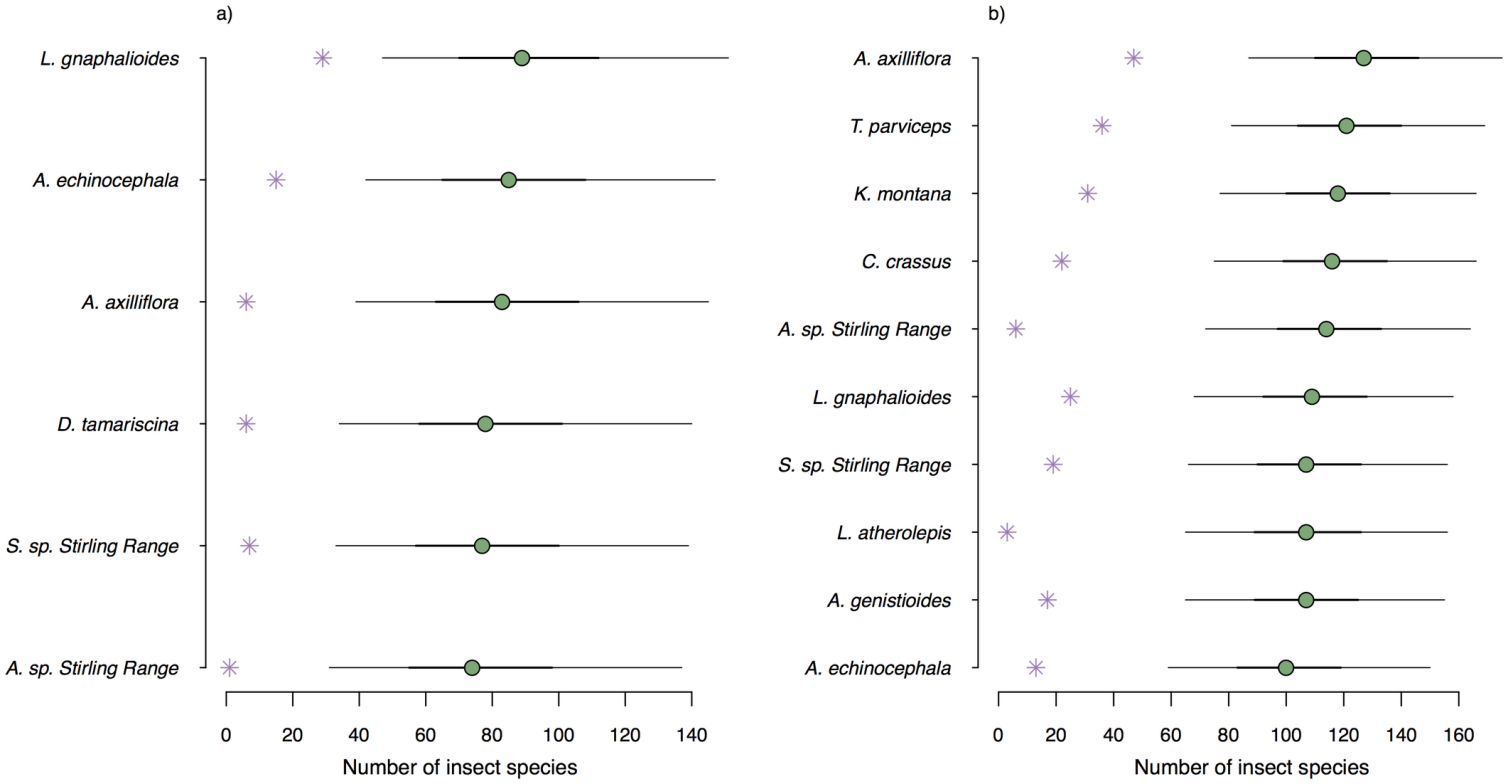
Number of insect species on each plant species in the Stirling Range case study. Purple stars show the observed number of insect species and green circles represent median estimates. Thick and thin black lines give 50% and 95% credible intervals, respectively.

The number of insect species that visited plant species was always estimated to be higher than in the observations (Fig 6). While median estimates ranged from 75-90 (95% CI 35-140) in 2012 and 101-127 (60-160) in 2013, observed numbers were much lower, 2-23 in 2012 and 4-47 in 2013. The credible intervals around the median estimate never included the observed number of animal species.

## Discussion

Here, we presented a modelling framework that accounted for imperfect detection and uncertainty to estimate the coextinction risk of species in mutualistic plant-animal networks and assess network structure. Interaction network studies are usually based on the crude assumptions of networks being static, well-represented through observed links, and reflecting true diet breadths of species [11, 19]. Our approach, using matrices of counts of animal species on individual plants, maintained the information about the distribution of interactions across plant individuals—information which is usually lost in aggregated species-by-species networks [21, 31]. In maintaining this information, we were able to separate the influences of an animal species ‘abundance and its preference for a plant species.

We applied our model to simulated networks and a case study network of flower-visiting insects and flowering plants, and found that model predictions were always as good as or better than observations, particularly for communities with high interaction rates and low abundances. We also predicted that a large number of visiting insects was missed in the sampling process and thereby provided additional evidence to previous concerns about the sampling completeness of communities [19, 21–23]. Even after a second field season with a larger observed species richness (factor of 2.5), the model predicted that 73-244 species remained undetected—a plausible result given that our study site was situated in a biodiversity hotspot with very high insect richness [50]. Finally, we showed that the model predictions improved with increasing sampling effort for low animal abundance.

Our case study network appears most similar to the HILA scenario with low sampling effort and is common for mutualistic networks [51]. Consequently, predicted numbers of interaction partners were predominantly higher than observed ones and exhibit considerable uncertainty. For insects with high abundances, observed and median predicted numbers of plant partners were very similar (e.g., *S. pacificus*, *Lasioglossum* sp.101). For empirical datasets, doubling or tripling the sampling effort would certainly increase model accuracy, as was visible by the reduction of uncertainty with increasing sampling size for low abundance scenarios. While increasing sampling effort might be possible for studies in easily accessible areas, in more remote areas it would be cumbersome and expensive. Extending our study threefold with the same number of observers, would have exceeded the flowering period of the focal species and increased the expenses considerably. Trippeling our personnel would have increased the risk of trampling flora and spreading the plant pathogen *P. cinnamomi*. Observational studies should consider combining increased sampling effort and applying predictive models like ours to analyse interaction networks that exhibit a similar structure as in the network in situations when interactions are common but abundance of animals is low (HILA scenarios).

Other networks might exhibit different structures: antagonistic networks, such as insect herbivores and host plants appear to be more similar to the LILA scenario [31]. For LILA scenarios, the median estimates and observed numbers of plant species partners frequently aligned, except for small sampling efforts, hence modelling should be considered when sampling effort is low. For medium and higher sampling efforts, sampled observations served as good estimates for coextinction risks. For all networks with high abundances (e.g., internal parasites in gut systems of mammalian hosts [52]), a low sampling effort seems to be sufficient.

Our hierarchical modelling approach was flexible to different network types, which we demonstrated by applying it to different parameter scenarios. While the current model structure was dependent on a plant individual by animal species structure several structural extensions are also possible. For example, one could include covariates that may influence a species’ specialisation, such as phylogenetic information [53], species traits [22], resource availability [15], environmental, temporal or spatial variables [53]. Phylogenetic relationships of host use exist for parasites [53] and herbivores [54], but it is unclear if the phylogeny of flower-visitors affects plant visitations [34] or not [22]. If available species abundances could also be incorporated into the modelling approach to increase model accuracy and precision, and to help distinguish tourists from rare species (false positive observations). Abundances can be measured for large species (e.g., avian frugivores through mist-netting or camera traps), but for small species this is more difficult. Further, trait information may inform the model about forbidden links [11] by indicating if the phenology or morphology of species prevent their interaction. While the great flexibility of our model allows various extensions, one should bear in mind that every extension increases model complexity and computational costs.

While our model is flexible towards model extensions, it is sensitive to the data that it is fitted to, yet the conclusions from our study remain robust. Firstly, the estimated numbers of interaction partners are similar, or closer to the truth than the observations. Secondly, many animal species remain undetected in the observation procedure and our model can estimate the number of missed animal species (with uncertainty). Our sampling design (which is common for mutualistic studies) makes it impossible to estimate the number of plant species that remain undetected, despite the possibility that other interaction partners may occur in the study region or beyond [31]. Thirdly, animals may under certain circumstances shift their interactions to other, less preferred plant species—a process known as host-switching [55, 56] or re-wiring [57]. A species’ potential number of interaction partners is therefore likely to be higher than what a model predicts.

## Conclusion

Inferences from interaction networks may be useful for species conservation and ecosystem management [58], but we have shown that observed interaction networks suffer from imperfect detection of links and species, and are usually vastly undersampled. Our results suggest that network analysis based on observed interaction networks—even if they are quantitative—are biased by these unobserved interactions. Conclusions about the conservation status of communities and implications from current network studies (e.g., [59]) might therefore not be meaningful [58]. To overcome these issues, two options emerge: we either substantially increase our sampling effort in network studies, or apply statistical tools that approximate the underlying structure of ecological networks. Yet, empirical studies are time consuming, heavily dependent on weather conditions, expensive [24] and can harm threatened communities. We are confident that our model, which acknowledges sampling effort and uncertainty, serves as a good first step towards assessing the coextinction risk of interacting species and for investigating the use of interaction networks in conservation science.

## Supporting information

S1 Table Area under the receiver operator curve (AUC) values for the binary interaction parameter. AUC values for the binary interaction parameter describe how well the model performance in identifying true positive compared to false positives. Numbers close to 1 indicate a high identification rate of true positives and thereby a high model performance.
(DOCX)

S1 Fig Comparison of simulated and estimated parameters for augmented networks. Comparison of simulated and estimated parameters for the *high interaction, low abundance* scenario (HILA) and low observation effort (20 observations) when animal species were augmented. The already existing 150 dependent species were augmented to form supercommunities of 250 (b and f), 300 (c and g) and 450 (d and h) animal species. Simulated abundances and mean estimated abundances (a-d) are presented with their 95% credible interval on a natural logarithmic scale. Binary simulated interactions are compared to mean estimated interaction and points are jittered for better visibility (e-h).
(TIFF)

S2 Fig Map of the study site’s location in south-west of Western Australia. The community of flower-visiting insect species on a threatened ecological plants was located on Bluff Knoll, the highest peak of the Stirling Range National Park (outlined shows National Park borders) in the south-west of Western Australia. The inset map gives the study site’s location in Australia.
(TIFF)

S3 Fig Comparison of true (x-axis) and estimated abundances (y-axis) for networks scenarios. Networks were simulated for high and low interaction probabilities and abundances and three sampling intensities (20, 40 and 60 observations). Order of scenarios: a-c) *high interaction probability, high abundance* (HIHA); d-e) *low interaction probability, high abundance* (LIHA); g-i) *high interaction probability, low abundance* (HILA); j-l) *low interaction probability, low abundance* (HILA). The grey line represents a perfect correlation between the two groups.
(TIFF)

S4 Fig Comparison of true interactions (x-axis) and mean estimated interactions (y-axis) in simulated scenarios. Networks were simulated for high and low interaction probabilities and abundances and three sampling intensities (20, 40 and 60 observations). We present overlying points jittered to increase their visibility. Order of scenarios: a-c) *high interaction probability, high abundance* (HIHA); d-e) *low interaction probability, high abundance* (LIHA); g-i) *high interaction probability, low abundance* (HILA); j-l) *low interaction probability, low abundance* (HILA).
(TIFF)

S5 Fig The cumulative distribution of links for animal and plant species against the number of links. The cumulative distribution of links for twelve simulated scenarios (blue), and heir corresponding sampled (purple) and 1000 predicted (green) networks. Order of scenarios: a-c) *high interaction probability, high abundance* (HIHA); d-e) *low interaction probability, high abundance* (LIHA); g-i) *high interaction probability, low abundance* (HILA); j-l) *low interaction probability, low abundance* (HILA).
(TIFF)

S1 Code R code for simulating interaction networks. (PDF)

S2 Code JAGS code for modelling interaction frequencies. (PDF)
