# Supplementary material for: Identifying species at coextinction risk when detection is imperfect: Model evaluation and case study
Source: PLoS One. 2017 Aug 28;12(8):e0183351. doi: 10.1371/journal.pone.0183351 (PMC5573280; doi:10.1371/journal.pone.0183351)
Supplement: S1 Table — AUC values for the binary interaction parameter describe how well the model performance in identifying true positive compared to false positives. Numbers close to 1 indicate a high identification rate of true positives and thereby a high model performance. (DOCX) [file pone.0183351.s001.docx]

| Scenario | Sampling intensity | | |
| --- | --- | --- | --- |
|  | 20 | 40 | 60 |
| hahu | 0.999868715 | 1 | 1 |
| halu | 0.999686716 | 0.99993895 | 1 |
| lahu | 0.874471184 | 0.953746233 | 0.971590719 |
| lalu | 0.863067444 | 0.920286646 | 0.965669054 |
