# Supplementary figures and images for: Identifying species at coextinction risk when detection is imperfect: Model evaluation and case study
Source: PLoS One. 2017 Aug 28;12(8):e0183351. doi: 10.1371/journal.pone.0183351 (PMC5573280; doi:10.1371/journal.pone.0183351)

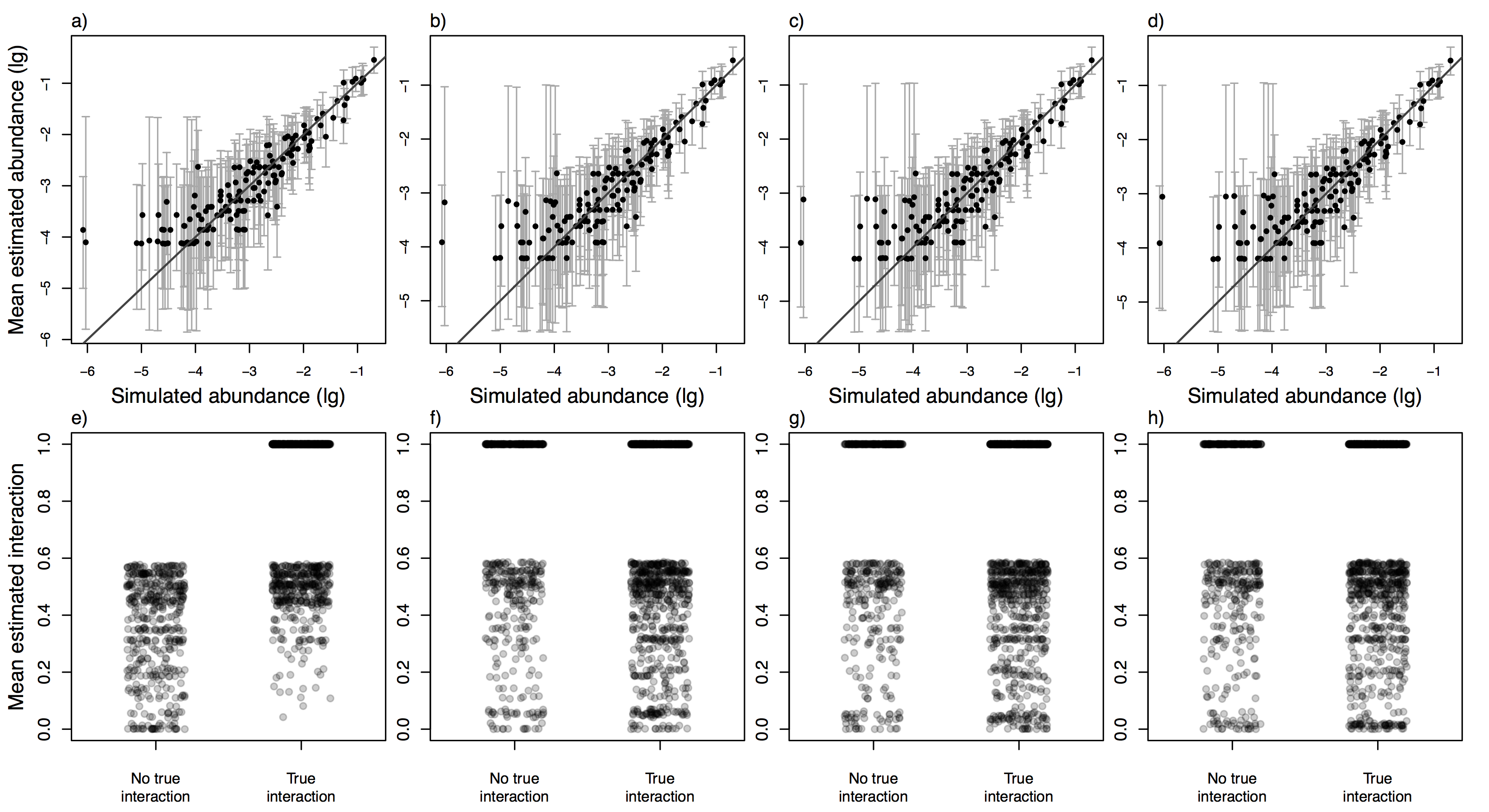

Supplement: S1 Fig — Comparison of simulated and estimated parameters for the high interaction, low abundance scenario (HILA) and low observation effort (20 observations) when animal species were augmented. The already existing 150 dependent species were augmented to form supercommunities of 250 (b and f), 300 (c and g) and 450 (d and h) animal species. Simulated abundances and mean estimated abundances (a-d) are presented with their 95% credible interval on a natural logarithmic scale. Binary simulated interactions are compared to mean estimated interaction and points are jittered for better visibility (e-h). (TIFF) [file pone.0183351.s002.tiff]

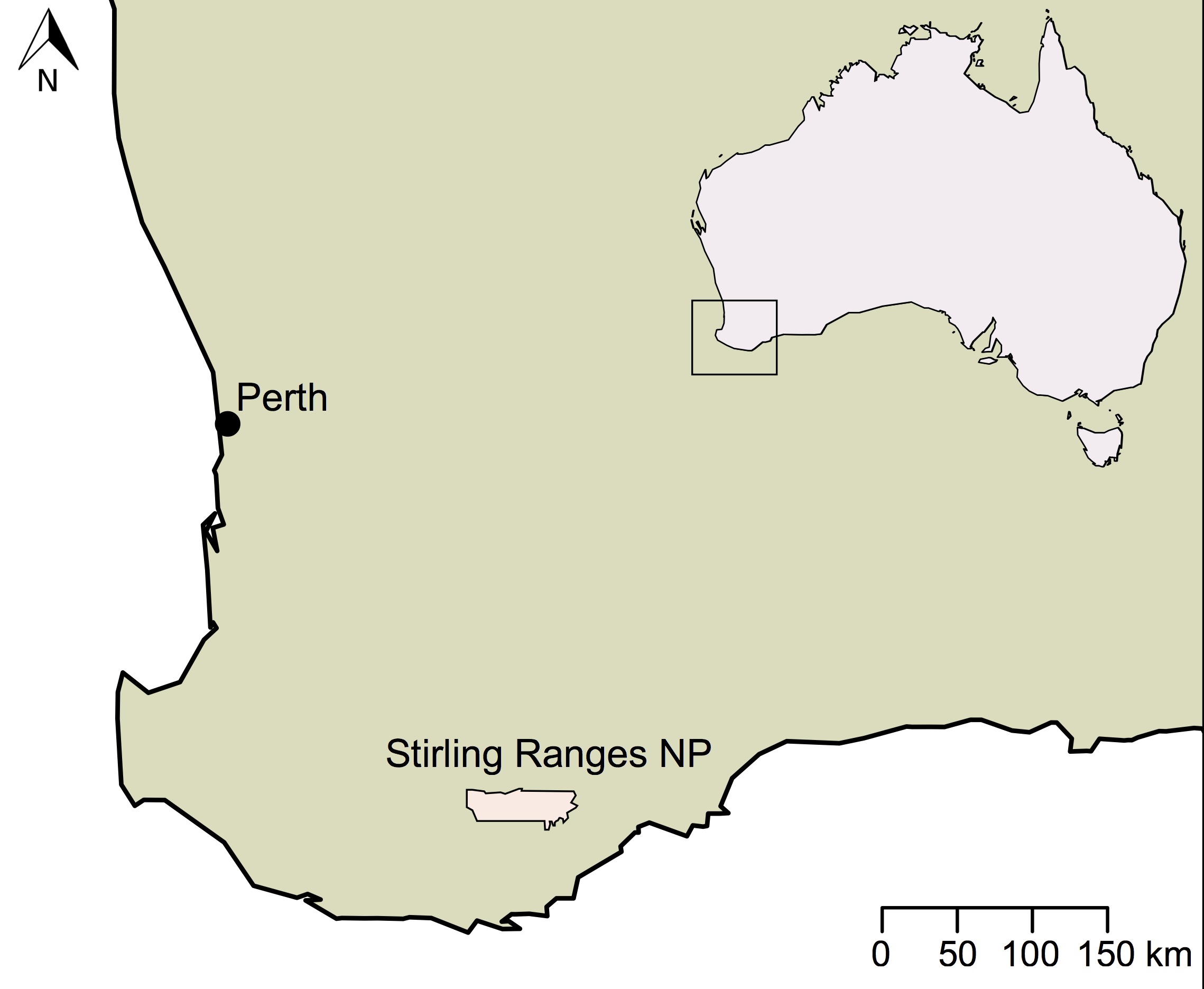

Supplement: S2 Fig — The community of flower-visiting insect species on a threatened ecological plants was located on Bluff Knoll, the highest peak of the Stirling Range National Park (outlined shows National Park borders) in the south-west of Western Australia. The inset map gives the study site’s location in Australia. (TIFF) [file pone.0183351.s003.tiff]

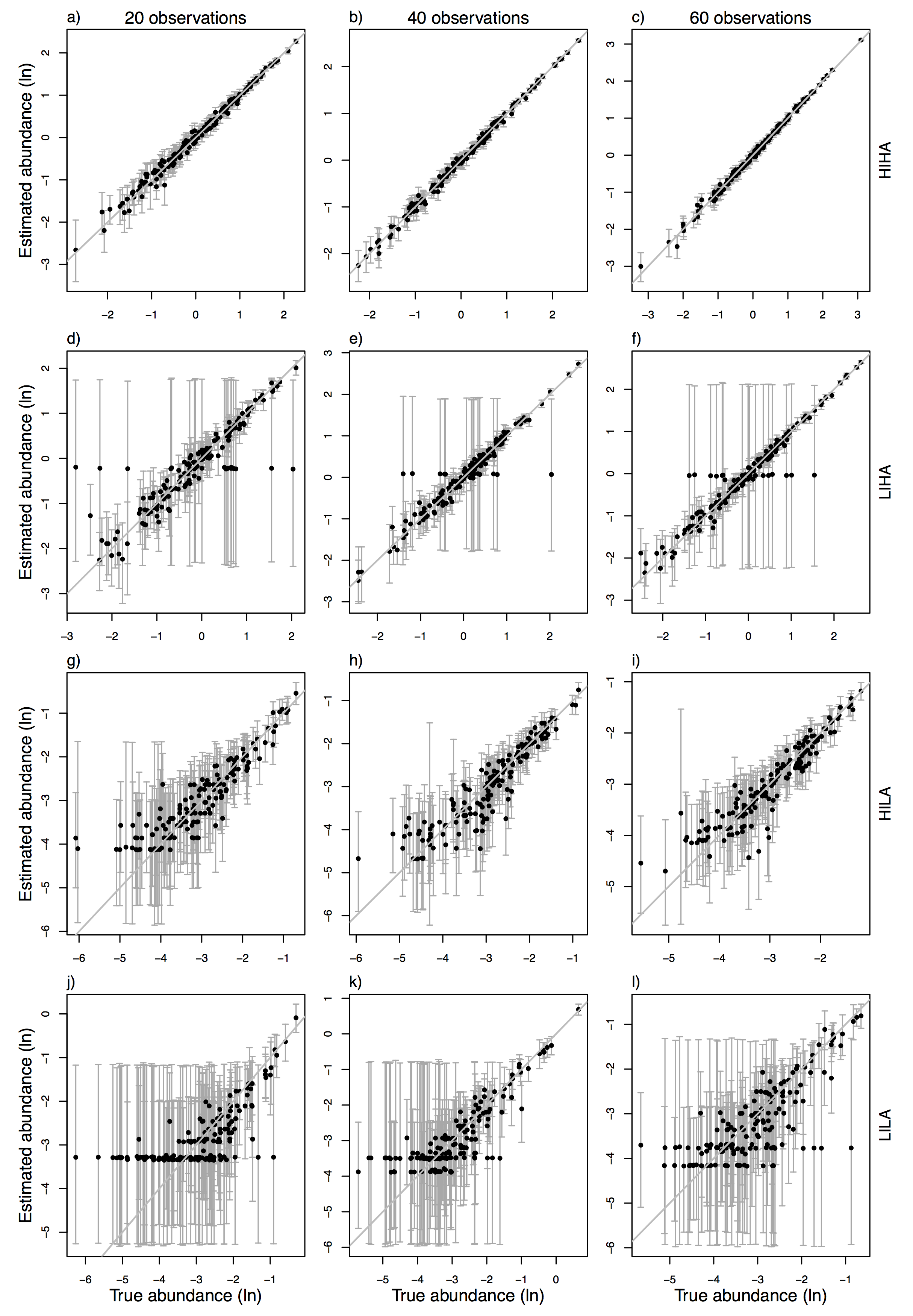

Supplement: S3 Fig — Networks were simulated for high and low interaction probabilities and abundances and three sampling intensities (20, 40 and 60 observations). Order of scenarios: a-c) high interaction probability, high abundance (HIHA); d-e) low interaction probability, high abundance (LIHA); g-i) high interaction probability, low abundance (HILA); j-l) low interaction probability, low abundance (HILA). The grey line represents a perfect correlation between the two groups. (TIFF) [file pone.0183351.s004.tiff]

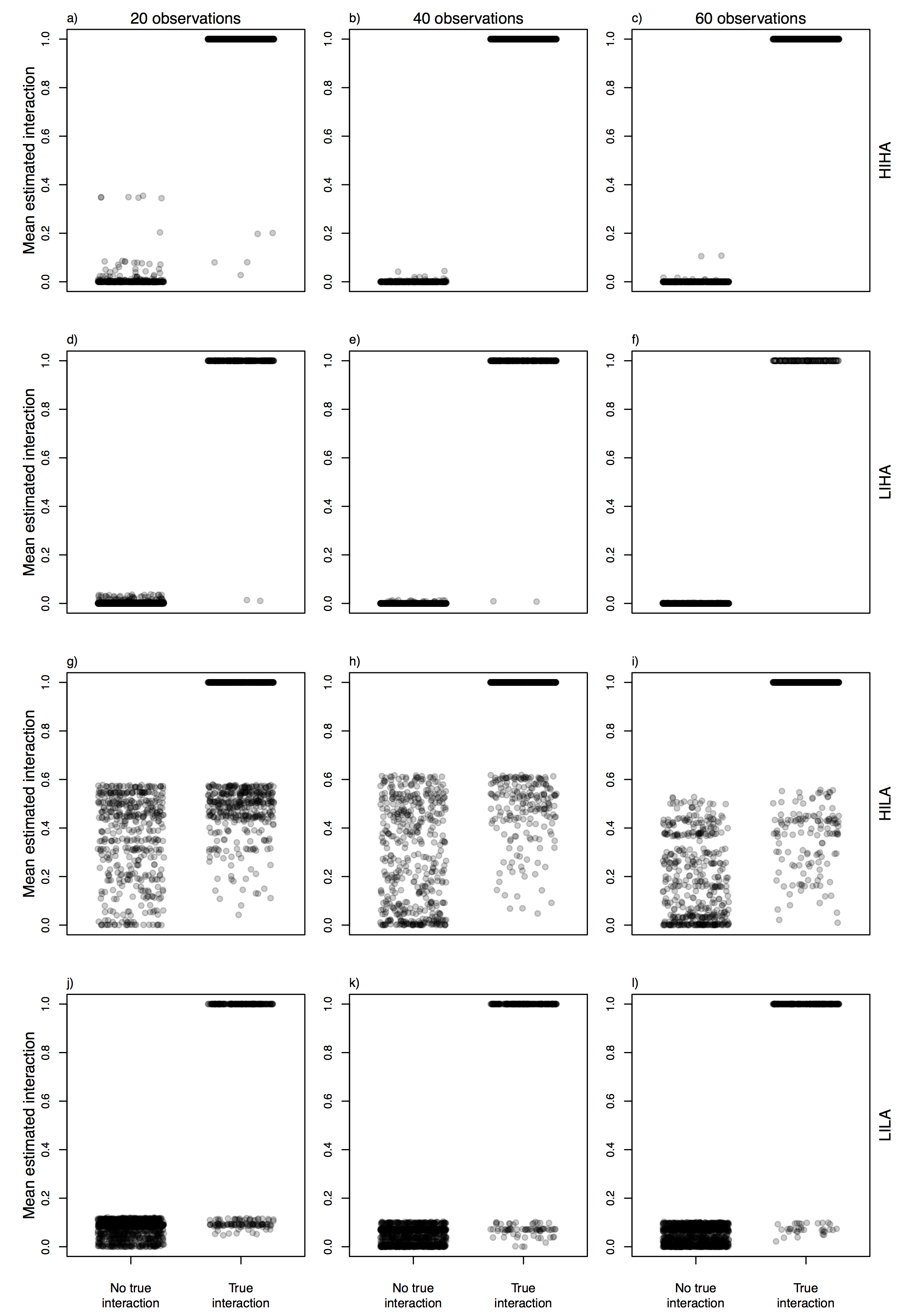

Supplement: S4 Fig — Networks were simulated for high and low interaction probabilities and abundances and three sampling intensities (20, 40 and 60 observations). We present overlying points jittered to increase their visibility. Order of scenarios: a-c) high interaction probability, high abundance (HIHA); d-e) low interaction probability, high abundance (LIHA); g-i) high interaction probability, low abundance (HILA); j-l) low interaction probability, low abundance (HILA). (TIFF) [file pone.0183351.s005.tiff]

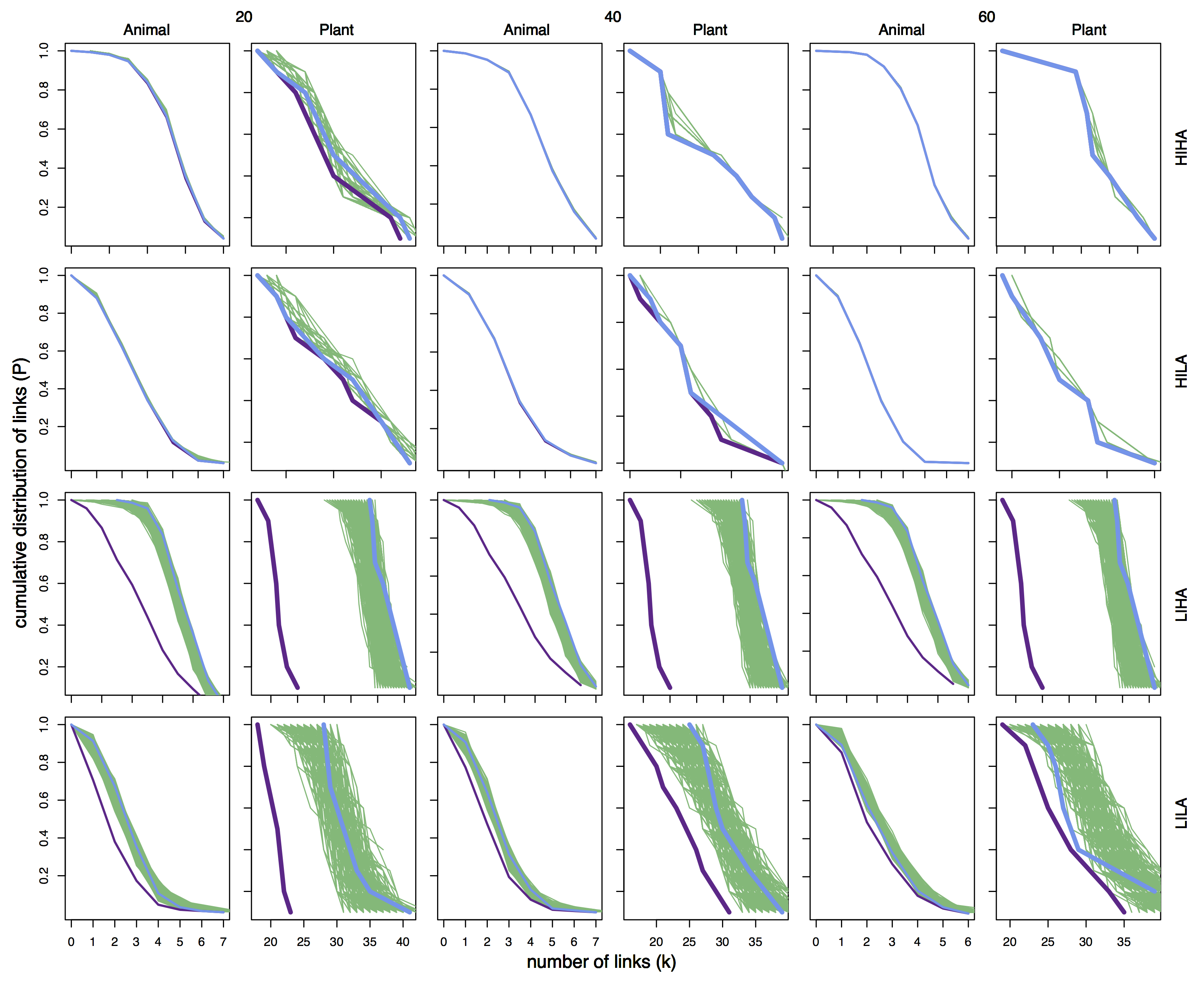

Supplement: S5 Fig — The cumulative distribution of links for twelve simulated scenarios (blue), and heir corresponding sampled (purple) and 1000 predicted (green) networks. Order of scenarios: a-c) high interaction probability, high abundance (HIHA); d-e) low interaction probability, high abundance (LIHA); g-i) high interaction probability, low abundance (HILA); j-l) low interaction probability, low abundance (HILA). (TIFF) [file pone.0183351.s006.tiff]
