# Supplementary material for: Identifying species at coextinction risk when detection is imperfect: Model evaluation and case study
Source: PLoS One. 2017 Aug 28;12(8):e0183351. doi: 10.1371/journal.pone.0183351 (PMC5573280; doi:10.1371/journal.pone.0183351)
Supplement: S1 Code — (PDF) [file pone.0183351.s007.pdf]

```
model { #start model code
  for (i in 1:Nobs) {
    # observed count on a host individual is drawn from a Poisson
with mean abundance
    count[i] ~ dpois(mu.lambda[i])

    # mean count = use of a host by a dependent sp. (theta) *
characteristic abundance (lambda)
    mu.lambda[i] <- theta[ins.sp[i], pl.sp[i]] * lambda[i]

     log(lambda[i]) <- beta[ins.sp[i]]   # backtransform
log(abundance)
   }  # end i loop round observations

   for (j in 1:(Nins)) { # for each dependent species
     w[j] ~ dbern(omega) # availability indicator variable
     HB_invert[j] <- sum(theta[j, ]) # Host breadth for each
dependent species

     for (k in 1:Npl.sp) { # for each host species
       theta[j, k] ~ dbern(lg_theta[j, k]) # use indicator
       lg_theta[j, k] <- psi[j, k] * w[j] # probability of
interaction conditional on dependents being available
       logit(psi[j,k]) <- alpha[j,k] # back transformation of grand
mean probability of interaction
       alpha[j,k] ~ dnorm(alpha_0, tau_a) # logit(probability of
interaction)
     } # end host species loop

     beta[j] ~  dnorm(beta_0, tau_b) # log(abundance)
   } # end dependent species loop

   for (k in 1:Npl.sp){ # host sp level
    ID_plant[k] <- sum (theta[,k]) # Insect diversity for each host
species
   } # end host individual loop


    alpha_mean ~ dunif(0, 1) # prior distribution for grand mean
probability of use
    alpha_0 <- log(alpha_mean) - log(1-alpha_mean) # probability of
interaction is estimated on a logit scale

    beta_mean ~ dunif(0, 10) # prior distribution for grand mean
abundance
    beta_0 <- log(beta_mean) # abundance on a log scale

    sigma_a ~ dt(0, 0.1, 1)T(0, ) # standard deviation for
probability of interaction
    sigma_b ~ dt(0, 0.1, 1)T(0, ) # standard deviation for abundance

    tau_a <- pow(sigma_a,-2) # precision for probability of
interaction
```

```
    tau_b <- pow(sigma_b,-2) # precision for abundance

    omega ~ dunif(0,1) # indicator variable that describes if
dependent sp is availability

    n0 <- sum(w [(n+1): (Nins)]) # sums all the non-available insect
    N <- n + n0 # species richness estimate

} # end model
```
