# Supplementary material for: Identifying species at coextinction risk when detection is imperfect: Model evaluation and case study
Source: PLoS One. 2017 Aug 28;12(8):e0183351. doi: 10.1371/journal.pone.0183351 (PMC5573280; doi:10.1371/journal.pone.0183351)
Supplement: S2 Code — (PDF) [file pone.0183351.s008.pdf]

```
# Scale parameters for hyper distributions
mu.interaction   <- 1.5  # mean interaction probability; or -2.5 (for low)
sd.interaction   <- 2.5  # standard deviation of interaction probability;
mu.abund <- -3  # mean abundance of dependent or 0 (for low)
sd.abund <- 1  # standard deviation of abundance of dependent

# Dimensions of simulated networks
n.dep   <- 150  # number of dependent species
n.host  <- 10  # host species
n.coll  <- 20  # number of observations per host species
dep.sp  <- rep(1: sum(n.dep), each = n.host * n.coll)
host.sp <- rep(rep(1: n.host, each = n.coll), times = sum(n.dep))
coll    <- rep(1: n.coll, rep((sum(n.dep) * n.host), n.coll))

# Create empty vectors
interaction.prob <- rep(NA, n.host * (sum(n.dep)))  # mean abund

abund <- rep(NA, times = sum(n.dep))  # mean

x <- rep(NA, times = (sum(n.dep) * n.host))  # logit-1 transformation of interaction
probability

x1 <- rep(NA, times = (n.host * sum(n.dep)))  # binary interaction indicator

host.sp.ind <- rep(NA, times = (n.host * sum(n.dep)))  # track host species identity

dep.sp.ind <- rep(NA, times = (n.host * sum(n.dep))) # tracks dependent species
identity

interaction <- rep(NA, times = sum(n.dep) * n.host * n.coll)  # realised interaction

exp.abund <- rep(NA, times = sum(n.dep) * n.host * n.coll) # expected abundance

host.sp.count.ind <- rep(NA, times = sum(n.dep) * n.host * n.coll)

dep.sp.cnt.ind <- rep(NA, times = sum(n.dep) * n.host * n.coll)


# Data simulation
for (i in 1:sum(n.dep)) {
  abund[i] <- rnorm(1, mean = mu.abund, sd = sd.abund)

    for (l in 1:n.host) {
      interaction.prob[i+(l - 1) * sum(n.dep) * n.host * sum(n.dep)] <- rnorm(1, mean =
mu.interaction, sd = sd.interaction)

      x[i+(l - 1) * sum(n.dep) * n.host * sum(n.dep)] <- exp(interaction[i + (l - 1) *
sum(n.dep) * n.host * sum(n.dep)]) / (1 + exp(interaction[i + (l - 1) * sum(n.dep) *
n.host * sum(n.dep)]))
```

```
      x1[i + (l - 1) * sum(n.dep) * n.host * sum(n.dep)] <- rbinom(1, 1, x[i + (l - 1) *
sum(n.dep) * n.host * sum(n.dep)])

      dep.sp.ind[i + (l - 1) * sum(n.dep) * n.host * sum(n.dep)] <- i
      host.sp.ind[i + (l - 1) * sum(n.dep) * n.host * sum(n.dep)] <- l

      for (m in 1:n.coll) {
        interaction[i + (l - 1) * sum(n.dep) * n.host * sum(n.dep) + (m - 1) * n.host *
sum(n.dep)] <- x1[i + (l - 1) * sum(n.dep) * n.host * sum(n.dep)]

        exp.abund[i + (l - 1) * sum(n.dep) * n.host * sum(n.dep) + (m - 1) * n.host *
sum(n.dep)] <- rpois(1, lambda = exp(abund[i]))

        dep.sp.cnt.ind[i + (l - 1) * sum(n.dep) * n.host * sum(n.dep) + (m - 1) * n.host *
sum(n.dep)] <- i

        host.sp.count.ind[i + (l - 1) * sum(n.dep) * n.host * sum(n.dep) + (m - 1) * n.host
* sum(n.dep)] <- l
      }
    }
  count <- exp.abund * interaction
}
```
